# Supplementary material for: Chromothripsis during telomere crisis is independent of NHEJ, and consistent with a replicative origin
Source: Genome Res. 2019 May;29(5):737–49. doi: 10.1101/gr.240705.118 (PMC6499312; doi:10.1101/gr.240705.118)
Supplement: Supplemental Material [file supp_gr.240705.118_Supplemental_file_1.zip › contigs/annotated_contigs/DB57/contig.2.DB57_length_485_mean_cov_5.56701030928.docx]

**DB57_length_485_mean_cov_5.56701030928**

```
ATAGGAGGAGAAAGAAGACAGAGTTACTCAGAAAAATCTATTTAGGTGGAGCAGAAACTGGCATTATTTTCCCCAGATCCTTTAGAAGC
 >chr4:19441473-19441661 + E=1e-98
AGCATGGCCCTGCTGATACCTTGAATTTTAATTTCTAGCCTTTATAACCGTAATATAATAATTTCTGTCATTTTAATCCACCGAGTTGT

GCTAATT|TGT|GTTACTGTGGTTACCAGAAGCTTCTCTCATTCCAGGAAGAATCTGCTTTCTGGGCTATAGAACGGGCTTTAAAATCA
        >chr4:19685333-19685633 + E=5e-169
GCAGGAACCAGACATGTTGAAGCAGAGACGAGTGAGGGGCACAAAGAACCTATGATTATTTTCAAAGCATCCTGGGAAATTCCAGGAAA

GATTCTGGAGTTTTCTTTCAAAATGATAACTTACGGACAGTTTGAGGAAAAGCTATGGAAATTAAATGATTTGATTTTCCTGCCATAGA

TAGAACAGTCACTTTTTATTCTACAGCTATCCTCCCCCACCT
```
